# Clinical utility of cerebrospinal fluid-derived circular RNAs in lung adenocarcinoma patients with brain metastases

**DOI:** 10.1186/s12967-022-03274-1

**Published:** 2022-02-05

**Authors:** Zhen Wang, Ruoying Yu, Xiaoxi Chen, Hua Bao, Ran Cao, An-Na Li, Qiuxiang Ou, Hai-Yan Tu, Qing Zhou, Xue Wu, Zhi-Bo Lin, Yi-Long Wu

**Affiliations:** 1 Guangdong Provincial People’s Hospital, Guangdong Academy of Medical Sciences, Guangdong Lung Cancer Institute, Zhong Shan 2nd road 106, Guangzhou, 510080 Guangdong China; 2 Geneseeq Research Institute, Nanjing Geneseeq Technology Inc., Nanjing, Jiangsu China; 3 Guangdong Cardiovascular Institute, Guangzhou, China

**Keywords:** Circular RNA, Cerebrospinal fluid, Lung ADC brain metastasis, Overall survival

## Abstract

**Background:**

Free circular RNAs(circRNAs) escaping from primary lesion of cancer to brain are strictly regulated by blood–brain barrier and therefore cerebrospinal fluid (CSF) circRNAs have potential advantage in exploring biomarkers and mechanism of brain metastasis in lung cancer.

**Methods:**

We collected paired cerebrospinal fluid, plasma and tumor tissues from 21 lung adenocarcinoma (ADC) patients with brain metastases (BM) and performed RNA sequencing.

**Results:**

Compared to tumor tissue and plasma, circRNAs in CSF were characterized by lower number of spieces but higher abundance. Notably, CSF-circRNAs displayed high heterogeneity among different BM lung ADC patients. A total of 60 CSF-circRNAs was identified and associated with shorten overall survival. The circRNA-miRNA-mRNA network analysis revealed that the 60 CSF-circRNAs involved in cancer-associated pathways, and five of them showed strong association with WNT signaling pathway. Validation by RT-PCR of CSF and in vitro experiments of the five candidate circRNAs support their potential roles in cell proliferation and invasion.

**Conclusions:**

In summary, our results depicted the heterogenous CSF-circRNAs profiles among BM lung ADC and implied that CSF-circRNAs may be promising prognosis-related biomarkers.

**Supplementary Information:**

The online version contains supplementary material available at 10.1186/s12967-022-03274-1.

## Introduction

Brain metastasis represents a great challenge among patients with lung cancer [1]. About half of brain metastasis in solid tumors were due to lung cancer [2, 3]. More than 40% of lung cancer develop brain metastases (BM) and the risk keeps increasing during their course of disease [1] and BM is usually associated with poor outcomes [1, 3, 4].

The difficulties in obtaining tumor tissue samples from BM largely preclude the molecular characterization and monitoring of disease [5]. Liquid biopsies exhibit advantages in molecularly characterizing brain metastatic cancers, however, circulating tumor DNA (ctDNA) in plasma have obvious limitations, *e.g.* limited detectable amount [6, 7]. Frequently renewed cerebrospinal fluid (CSF) keeps close contact with brain malignancies [8, 9] therefore provides a better solution as reservoir of tumor-related/secreted molecules [5, 10]. On this basis, simultaneously profiling the molecular characteristics of CSF, tumor tissues and plasma would be of great help in exploring the pathogenesis of brain metastasis in lung cancer.

Different from DNA biomarkers, RNA can provide dynamic insights into the tumor states and regulatory processes [11]. Circular RNAs (circRNAs) generated by back-splicing involve in tumor pathogenesis through mechanisms such as microRNA (miRNA) sponges, combining RNA binding proteins etc. to regulate transcription and translation [12] in a wide variety of cancers including hepatocarcinoma [13], lung [14], gastric [15], colorectal [16], breast [17], and other cancers [18]. Some specific circRNAs present as promising biomarkers or therapeutic targets [18].

Free circRNAs escaping from primary solid tumors (*e.g.* lung cancer) to the brain is strictly regulated by presence of blood–brain barrier [19]. CSF-circRNAs could facilitate investigation due to their high abundance and conservation in the brain [20, 21]. CircRNAs commonly shared between primary and brain metastatic tumors are likely to account for some mechanism underlying lung cancer BM as well as be potential biomarkers.

Most studies specified circRNAs in certain type of tissues [13–17, 22–25] and seldom compared circRNAs across samples (*e.g.* cerebrospinal fluids, plasma, tumor tissues) [26]. In this study, we profiled circRNAs in 21 lung cancer patients with brain metastases by comparing paired samples including cerebrospinal fluid, plasma, and tumor tissue. A total of 60 candidates lung cancer-associated CSF-circRNAs were identified as close correlation with clinical outcomes. Bioinformatics analysis and preliminary validation concentrated on five circRNAs in the top-ranked Wnt signaling pathway supporting their possible roles in cell proliferation and invasion. Our data revealed CSF-circRNAs might be promising prognosis-related biomarkers in the lung ADC patients with brain metastases.

## Materials and methods

### Patient sample collection

Patients diagnosed with lung adenocarcinoma by histology and BM by magnetic resonance imaging (MRI) were recruited. Peripheral blood, tumor tissue and CSF were collected for pathological and genetic analysis. The study was approved by local independent ethics committees and complied with the Declaration of Helsinki and Good Clinical Practice principles and written informed consent forms were obtained.

#### Targeted generation sequencing and data processing

DNA extraction, library preparation and targeted enrichment were carried out as previously described [27, 28]. In brief, genomic DNA from whole blood were extracted using the DNeasy Blood & Tissue Kit (Qiagen) according to the manufacturer's protocols. FFPE samples were de-paraffinized with xylene followed by genomic DNA extraction using QIAamp DNA FFPE Tissue Kit (Qiagen) following the manufacturer's instruction. CSF was obtained through lumbar puncture as a standard practice for lung cancer patients with brain metastasis following National Comprehensive Cancer Network (NCCN) guidelines. Within 4 h of collection, the cellular fraction was removed by two-step centrifugation at 4 °C including 1900*g* for 10 min then 16,000*g* for 10 min. Plasma samples were first separated with blood cell sediment after centrifuging whole blood samples at 4 °C for 10 min 1600*g*, then recentrifuged at 4 °C 16,000*g* for 10 min. Both cfDNA from CSF and plasma were extracted using Qiagen QIAmp Circulating Nucleic Acid Kit (Qiagen, Germany) following the manufacturer’s protocols. Genomic DNA from tumor tissue samples was extracted using QIAmp DNA FFPE Tissue Kit (Qiagen, Germany) following the manufacturer’s protocols. Plasma sample was first centrifuged at high speed to remove any cell debris, followed by cfDNA extraction from the supernatant using QIAamp Circulating Nucleic Acid Kit (Qiagen). Sequencing libraries were prepared using the KAPA Hyper Prep Kit (KAPA Biosystems) according to manufacturer's suggestions for different sample types. In brief, cfDNA or fragmented genomic DNA underwent end-repairing, A-tailing and ligation with indexed adapters sequentially, followed by size selection using Agencourt AMPure XP beads (Beckman Coulter). Hybridization-based target enrichment was carried out with pan-cancer gene panel of 139 cancer-relevant genes, and xGen Lockdown Hybridization and Wash Reagents Kit (Integrated DNA Technologies). Captured libraries by Dynabeads M-270 (Life Technologies) were amplified in KAPA HiFi HotStart ReadyMix (KAPA Biosystems) and quantified by qPCR using the KAPA Library Quantification Kit (KAPA Biosystems) for sequencing.

The libraries were paired-end sequenced on NGS platforms (Illumina) according to the manufacturer's instructions. Trimmomatic [29] was used for FASTQ file quality control (below 15 or N bases were removed). Reads were then mapped to the reference Human Genome (hg19) using Burrows-Wheeler Aligner (BWA-mem, v0.7.12; https://github.com/lh3/bwa/tree/master/bwakit). Local realignment around the indels and base quality score recalibration was applied with the Genome Analysis Toolkit (GATK 3.4.0; https://software.broadinstitute.org/gatk/), which was also applied to detect germline mutations. VarScan2 [30] was used for somatic mutation detection. Somatic variant calls with at least 0.2% mutant allele frequency (MAF) and with at least three supporting-reads from both directions were retained. Common SNPs were filtered out using dbSNP (v137) and the 1000 Genomes database, followed by annotation using ANNOVAR [31]. Genomic fusions were identified by FACTERA [32] with default parameters. Copy-number variations (CNVs) were detected using ADTEx (http://adtex.sourceforge.net) with default parameters. Somatic CNVs were identified using paired normal/tumor samples for each exon with the cut-off of 0.65 for copy-number loss and 1.50 for copy-number gain.

### RNA-sequencing

The RNA-sequencing was performed following previous report [33]. Tumor tissue total RNA was extracted with miRNeasy mini-Kit (Qiagen, Hilden, Germany) following instructions, which concentration was assessed using NanoDrop-100 (Thermo Fisher Scientific, Wilmington, DE, USA) and Qubit RNA HS Assay Kit with Qubit 3.0 fluorometer (Life Technologies). Quality control (QC) process was completed via the Agilent 2100 bioanalyzer. Sequencing libraries were constructed with 1000 ng total RNA using KAPA RNA HyperPrep Kit and RiboErase (KAPA Biosystems, Wilmington, MA). cDNA was synthesized after ribosomal RNA depletion and fragmentation for 6 min at 94 °C. Prior to sequencing on HiSeq4000 platform, libraries were examined for quality and quantity with KAPA Library Quantification Kit (KAPA Biosystems) using qRT-PCR (CFX384 real time system, Bio-Rad Laboratories).

Plasma and CSF samples stored at − 80 °C were centrifuged at 16,000*g* for 10 min. The supernatants were transferred to new tubes for RNA extraction. Total RNA was isolated using Norgen Plasma/Serum Circulating and Exosomal RNA Purification Mini Kit (NorgenBiotek, ON, Canada), which concentration was determined using NanoDrop-100 (Thermo Fisher Scientific, Wilmington, DE, USA) and Qubit microRNA Assay Kit. Sequencing libraries were generated using SMARTer Stranded Total RNA-Seq Kit v2-Pico Input Mammalian (Pico v2, total RNA, Takara). Sequencing was performed on Illumina Hiseq 4000 machine.

### Data processing

The RNA-Seq reads passing queue thresholds were trimmed of adaptor sequences and aligned to the GRCh37 reference genome with Gencode (v24lift37) [33] annotation using STAR (v2.5.3) [33]. RNA expression abundance was quantified with raw counts from STAR by setting quantMode for GeneCounts parameter. Trimmed Means of M-Values (TMM) normalization was performed on the library size adjusted read counts, which was further converted to FPKM with edgeR (v3.16.5) [33]. Total exon length was calculated using the GenomicFeatures package [34] and used in the FPKM calculation. The number of circRNA species was defined as the number of circRNAs with read counts > 0. The QC information of samples was shown in Additional file 1: Table S1. The median number of circRNA reads was 4024, which was similar to the reported range from other studies [35, 36]. The median percentage of total reads mapping was 89.34%. The median percentage of high-quality (HQ) reads were 97.90%. The median of HQ %Q30 and raw reads %Q30 were 94.03 and 90.64 respectively, suggesting the reliability of the next-generation sequencing results.

### Circular RNA identification

The back-splicing reads were extracted from STAR results (chimeric.out.junction) and annotated using CIRCexplorer (v2.3.0) [33]. The identified CircRNAs for each sample were combined together and back-splicing reads numbers were extracted for the abundance prediction. Consistent with previous report [33], the effective length for circRNA was twice the read length less twice the anchor-size since only reads on this range could be unbiased evidence supporting circRNA. On this basis, FPKM was calculated using edgeR(v3.16.5) [33] and normalized with library sizes and normalization factors from linear transcripts for the same sample. Multiple circRNAs generated from the same gene were treated separately. Principal component analysis (PCA) was performed with the FPKMs of circRNAs (Additional file 2: Table S2) from different sample types. The samples from different batches (a, b, c, d, e, f) are clustered together and not clustered according to the batches, suggesting that the batch effect was limited (Additional file 6: Figure S1).

### Survival analysis

Cox proportional hazards model in R Survival package (v2.44–1.1) was used to identify independent predictors of survival either without (univariate) or with (multivariate) adjustment for relevant clinical covariates (age, gender, smoking history, metastasis). Patient groups were dichotomized by the expression of each circRNA or the median number of detected circRNA species in the 60 candidates list (circRNA-low: less than median, circRNA-high: more than median). The Kaplan–Meier survival curves were constructed using R Survminer package (v0.4–4) and the log-rank test was used to compare survival time between groups.

### Construction of circRNA-miRNA-mRNA network

One of the first and most investigated functions of circRNAs is that of a miRNA sponge that circRNAs binds to miRNAs and consequently represses their function [37]. Since circRNA are created by back-splicing events from linear mRNA and the miRNA sponge process is similar to miRNA-mRNA interaction, so we used miRanda software [38], a miRNA target scanner that aims to predict mRNA targets for microRNAs, to predict the interacted circRNA-miRNA interaction. First, miRNA-binding sites in circRNAs were extracted from the 5′ and 3′ ends (30 nt) and reverse-joined to produce a backsplice junction. Then the list of miRNAs that potentially bind to the given circRNA is generated by miRanda software using mature miRNA sequences from miRBase. To reduce the number of predicted miRNA binding sites, the parameter ‘-strict’ is applied when using miRanda [39]. The threshold miRanda-type score was set as greater than 170, and energy was set as less than − 30 with other default parameters. Finally, a total of 48 circRNAs and associated mRNAs and miRNAs were included. Then the miRNA-mRNA interaction was delineated with miRwalk [40] with default parameters. The graph of the circRNA-miRNA-mRNA network was drawn with the help of Cytoscape 3.7.1 [41]. The circRNAs, miRNAs and mRNAs of the circRNA-miRNA-mRNA network were shown in Additional file 3: Table S3. For the 5 annotated with the top-rank Wnt signaling pathway, Wnt pathway-related miRNAs and corresponding target genes were selected for each circRNA and used to generate the graphic interacting network.

### Co-expression of circRNA-mRNA and gene enriched pathways

To determine the association between the expression of circRNA and the regulation of mRNA co-expression, the Pearson correlation coefficient (PCC) between 60 selected circRNA and mRNA were compared. The absolute value of parameter PCC ≥ 0.70 and P value ˂ 0.01 was accepted and reserved for pathway analysis. A total of 52 circRNAs were included. The co-expressed mRNAs were shown in Additional file 4: Table S4.

### Annotation of circRNA co-expressed and target genes enriched pathways

GO and KEGG analysis of the co-expressed genes corresponding to the 60 circRNAs. The corresponding gene numbers were counted for Gene Ontology (GO) and Encyclopedia of Genes and Genomes (KEGG) pathway analysis, and significant correlations between target genes and their associated functions and pathways were assessed based on a threshold of P < 0.05. The function of target genes of 60 circRNAs based on circRNA-miRNA-mRNA network were annotated using Database for Annotation, Visualization and Integrated Discovery (DAVID) [42]. For clarity, functional annotations for target genes were focused on pathways in cancer. Finally, top-ranked signaling pathways and biological processes were selected on the basis of either gene numbers or p-values in each cluster.

### Lentiviral infection and cell proliferation

Lentiviruses used for circRNAs knockdown and the corresponding negative controls were purchased from Genechem. Short hairpin RNA (shRNA) targeting NOB1 gene was used as positive control (shPC) and the target sequence was AGGAGGAGGAGGAGGAAGAAA. The lentiviral vectors were separately transfected into A549 and HCC827 cells. Infection efficiency after 48–72 h was determined by GFP expression observed under a fluorescence microscope. Then the cells were harvested for cell proliferation analysis on a daily basis from day 1 to day 5 using Celigo Cell Cytometer (Nexcelom, USA). All experiments were performed in duplicates and repeated at least three times independently.

### Real-time PCR quantification

Total RNA was extracted using the SuperfecTRI, Total RNA Isolation Reagent (Trizol, Pufei, China). One μg total RNA was used for cDNA synthesis with M-MLV reverse transcriptase (Promega, USA). RT-qPCR was performed using SYBR Master Mixture (TAKARA, China). Primers were purchased from Ribobio (http://www.ribobio.com/). Results were normalized against *GAPDH* expression.

### MTT cell proliferation assay

The proliferation activity of cells was measured by an MTT assay. The cells (average 2 × 10^3^ cell/well) were seeded into 96-well plates with DMEM medium containing 10% FBS. MTT solution (5 mg/mL, 20 µL) was added to each well and the plates were incubated at 37 °C for 4 h and the supernatant was removed. After dissolving the formazan crystals in 100 µL of DMSO for five minutes, the absorbance of each well was measured using a spectrophotometer (Thermo Fisher Scientific, Vantaa, Finland) at 490/570 nm.

### Invasion assays

For invasion assays, A549 cells transfected with different shRNAs were seeded into the upper chamber of the Transwells (Corning, sigma-aldrich, USA) according to the manufacturer’s instructions. Medium containing 10% FBS was added to the lower chamber for 24 h. Cotton swabs were used to remove the non-invading cells that remained in the top chamber, and the cells that had invaded to the underside of the membrane were fixed in 4% paraformaldehyde for 30 min and stained with 0.5% crystal violet. Finally, images were obtained under a light microscope at 100X lens and 200X lens.

### Colony formation assay

Colony formation was performed as described previously [43]. A total of 4 ~ 10 × 10^2^ shRNA-transfected A549 cells suspended in DMEM medium containing 10% fetal bovine serum were plated in 6-well plates. Each well was done in triplicate. The plates were incubated at 37 °C in a 5% CO2 incubator for 14 days or until colonies with more than 50 cells. The cells were then fixed with 4% polyoxymethylene and stained with crystal violet. The experiments were done at least three times.

### RT-PCR and NGS validation

The primers for RT-PCR were designed according to the sequences of circRNA junction sites. Cell-free RNAs from CSF were extracted using Norgen Plasma/Serum Circulating and Exosomal RNA Purification Mini Kit (Slurry Format) according to the manufacturer’s instructions. cDNAs were synthesized using the SuperScript VILO Master Mix and used as templates for PCR amplification using Invitrogen™ Platinum™ SuperFi™ PCR Master Mix. Then the PCR products were subjected to agarose gel electrophoresis and PCR product libraries were constructed with cDNA segments extracted from gel using KAPA Hyper Prep C Kit according to the manufacturer’s instructions. Lastly, NOVAseq NGS was performed and the sequencing results were compared to the sequence of each circRNA junction site respectively.

### Statistical analysis

Statistical analyses were performed with the R package (v3.4.4). The Wilcoxon signed-rank test was used to analyze differences regarding the expression level of circRNAs and mRNAs. Pearson’s Coefficient was applied to compare the circRNA data and clinical results. *P* < 0.05 was set to be statistically significant.

Overall survival was defined from the diagnosis of brain metastases to death from any cause or last follow-up. Median follow up time was calculated using reverse Kaplan–Meier, which was obtained by reversing the event indicator so that the outcome became being censored. The Kaplan–Meier survival curves were constructed using R Survminer package (v0.4–4) and the log-rank test was used to compare survival time between groups.

Cox proportional hazards model in R Survival package (v2.44–1.1) was established to identify independent predictors of survival either without (univariate) or with (multivariate) adjustment for relevant clinical covariates (age, gender, smoking history, metastasis).

Patient groups were dichotomized by the expression of each circRNA or the median number of detected circRNA species in the 60 candidates list (circRNA-low: less than median, circRNA-high: more than median).

## Results

Between May 14, 2018 and October 26, 2015, a total of 21 lung adenocarcinoma (ADC) patients with brain metastases were selected. Patients’ clinicopathological data including sex, smoking status, specimen type, genomic alterations (*EGF, ERBB2, ALK, KRAS, TP53*) and treatment regimens were summarized (Table 1 and Additional file 5: Table S5). The median age was 55 years old, ranging from 39 to 74. There were 12 (57%) female and 9 (43%) male patients. Around 71% of patients were non-smokers while the rest (29%) were smokers. The size and number of BM lesions were available in 20 patients. Combining CSF cytology, brain MRI image, and symptoms, five patients were considered to have leptomeningeal carcinomatosis (LMC) as well. Eighteen patients had other non-cranial organ metastases including bone, lung, pleura, liver, adrenal gland (Additional file 5: Table S5). Subsequent treatment received in this cohort were TKI (9, 43%), TKI and stereotactic radiosurgery of BM (3, 14%), TKI and resection of BM (1, 5%), TKI and bevacizumab (1, 5%), chemotherapy (3, 14%), whole brain irradiation and best support care (2, 10%), best support care (2,10%) (Table 1 and Additional file 5: Table S5). A total of 15 tumor tissues, 19 CSFs, and 21 plasmas were sampled. Tumor tissues included primary tumors (5) and metastatic tumors from lymph node (7), lung (1), brain (1), pleura (1). Activating mutations were identified in tumor samples including *EGFR* L858R, *EGFR* T790M, *EGFR* exon 19 deletion, *ERBB2* amplification, *ALK* fusion, *KRAS* G12C.

**Table 1:** Patient demographic and clinical characteristics

Character	N (%) Total N = 21
Age at baseline, years	
Median	55
Range	39–74
Sex	
Male	9 (43%)
Smoking status	
Current smoker	1 (5%)
Former smoker	5 (24%)
Never smoker	15 (71%)
Activating mutation	
*EGFR* mut	17(81%)
*ALK* rearrangement	3 (14%)
*KRAS* mut	1 (5%)
LMC	
Yes*	5 (24%)
Baseline PS	
0	1 (5%)
1	19 (90%)
2	1 (5%)
Treatment received	
TKI**	9 (43%)
TKI + SRS of BM	3 (14%)
TKI + resection of BM	1 (5%)
TKI + bev	1 (5%)
Chemotherapy	3 (14%)
Pem + CBP + Bev	1 (5%)
Pem + Bev	1 (5%)
Paclitaxel + CBP + Bev	1 (5%)
WBI + BSC	2 (10%)
BSC	2 (10%)

*EGFR* epidermal growth factor receptor, *ALK* anaplastic lymphoma kinase, *KRAS* Kirsten rat sarcoma viral oncogene homologue, *mut* mutant, *BM* brain metastasis, *LMC* leptomeningeal carcinomatosis, *PS* performance status, *TKI* tyrosine kinase inhibitor, *SRS* stereotactic radiosurgery, *Pem* pemetrexed, *CBP* carboplatin, *Bev* bevacizumab, *WBI* whole brain irradiation, *BSC* best support care

^*^Patient was considered to have LMC should had at least one positive result of cerebrospinal fluid (CSF) cytology and brain magnetic resonance imaging (MRI). Three patients were diagnosed with LMC by CSF cytology, brain MRI, accompanied by neurological symptoms, which included headache, confusion, cognitive impairment and psychiatric disorders. The other two patients were diagnosed with LMC by brain MRI without symptom or CSF cytology

^**^Details of TKI regimens are shown in Additional file 5: Table S5

### The heterogeneity of CSF-circRNA in lung cancer

**Fig. 1:**
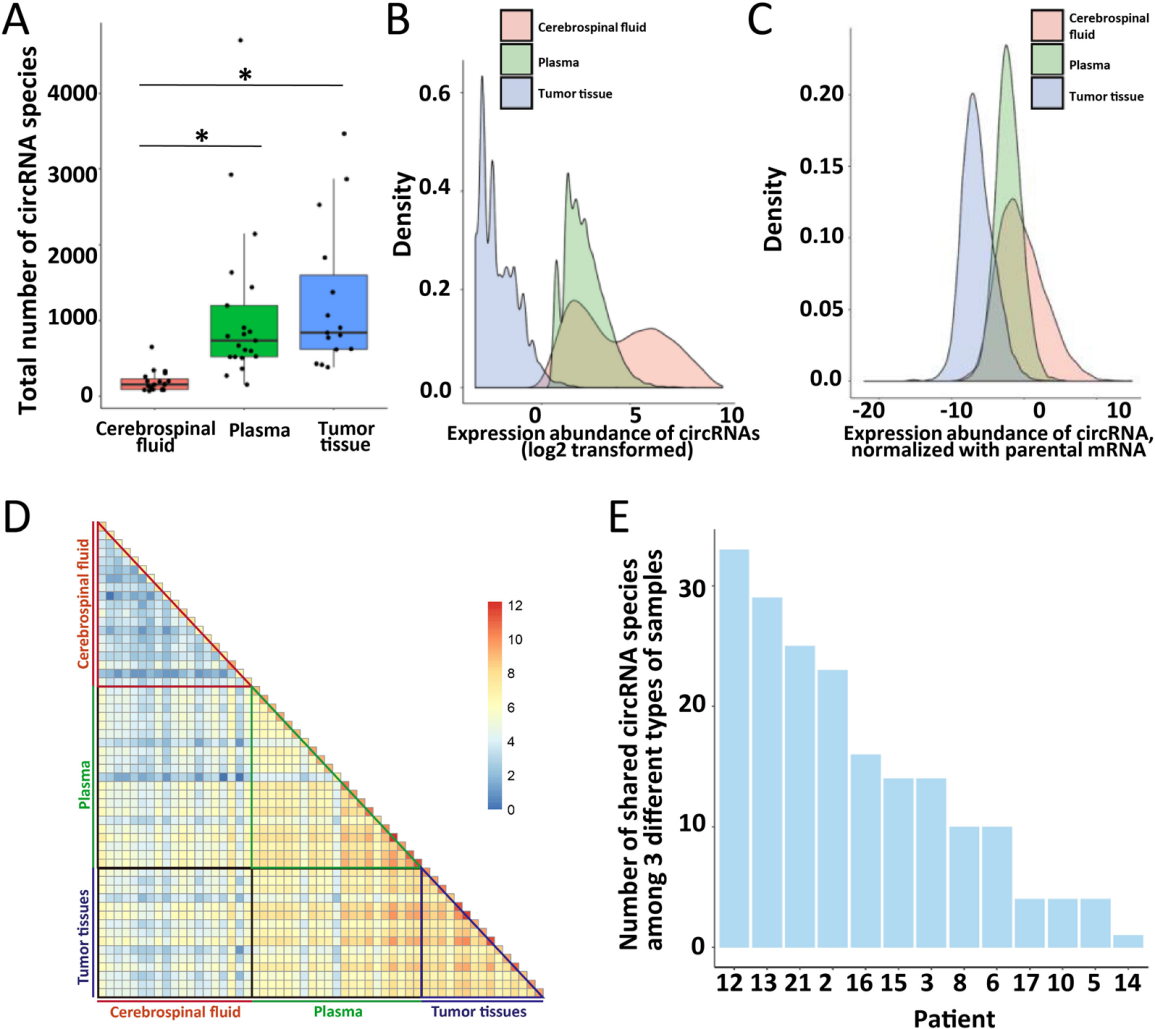
RNA profiling with cerebrospinal fluid, plasma, and tumor-tissue samples in lung cancer patients with brain metastasis. **A** Comparison of the number of detected circRNAs in cerebrospinal fluid, plasma, and tumor-tissue samples (*: p-value < 0.05). **B** The distribution of expression abundance of detected circRNAs in cerebrospinal fluid, plasma, and tumor-tissue samples. **C** The distribution of the ratio of expression of detected circRNAs to their parental mRNAs in cerebrospinal fluid, plasma, and tumor-tissue samples. **D** Log2-transformed number of shared circRNAs between different samples from the same or different patient(s) (patient 1 and 20: RNA-seq data not available for cerebrospinal fluid samples, patient 4, 7, 9, 11, 18 and 19: RNA-seq data not available for tumor-tissue samples). **E** Number of shared circRNAs among all 3 different samples (cerebrospinal fluid, plasma, and tumor-tissue) for individual patient

Total rRNA depleted RNA was sequenced for profiling circRNA in CSF, plasma and tumor tissue. The total number of circRNA species detected in CSF was significantly less than in plasma and tumor samples (*p* < 0.05, Fig. 1A), while the expression abundance of circRNAs in CSF was much higher (Fig. 1B and Additional file 6: Figure S2) even when normalized with parental mRNAs (Fig. 1C and Additional file 6: Figure S3). There was no significant correlation between expression abundance of circRNAs and their parental mRNAs among all the patients across sample types (Additional file 6: Figure S4). The number of commonly shared circRNA species among all three types of samples varied in patients (Fig. 1E and Additional file 6: Figure S5). Less shared circRNA species were found in CSF than in tumor tissue and plasma among patients (Fig. 1D), revealing high inter-patient heterogeneity of CSF. Within the individual patient, less shared circRNAs were found between tumor tissue and CSF than between tumor tissue and plasma (bottom, Fig. 1D), implying that circRNAs profile in CSF might bear more resemblance to that in brain metastatic tumors rather than in non-cranial tumors.

### Clinical characteristics and circRNA

Total circRNA species and CSF-circRNAs abundance displayed a trend of increase along with lines of therapies in BM lung cancer patients (*p* = 0.26, *p* = 0.66 Additional file 6: Figure S6), indicating a potential association between CSF-circRNAs and clinical outcomes. CSF-circRNA expression had a significant negative correlation with the CSF-ctDNA concentration (*r* = − 0.76, *p* = 0.028, Additional file 6: Figure S7). The trend of negative correlation between circRNA and ctDNA was also found in tumor tissue and plasma but without significance (tumor tissue: *r* = − 0.34, *p* = 0.23; plasma: *r* = − 0.21, *p* = 0.42, Additional file 6: Figure S7). Clinical features including sex, smoking status, and genomic alterations such as *EGFR/KRAS/TP53/ALK* mutations were not associated with total counts or abundance of CSF-circRNAs (Additional file 6: Figure S8, CSF). In plasma, circRNA showed significantly higher expression abundance in mutant *TP53* than in wild type. The total number of circRNA species but not expression abundance was higher in plasma from male patients and in tumor tissue from non-smokers and *ALK* mutations carriers (Additional file 6: Figure S8, plasma and tissue) than in their according counterpart.

### Identification of CSF-circRNAs correlated with clinical outcome

**Fig. 2:**
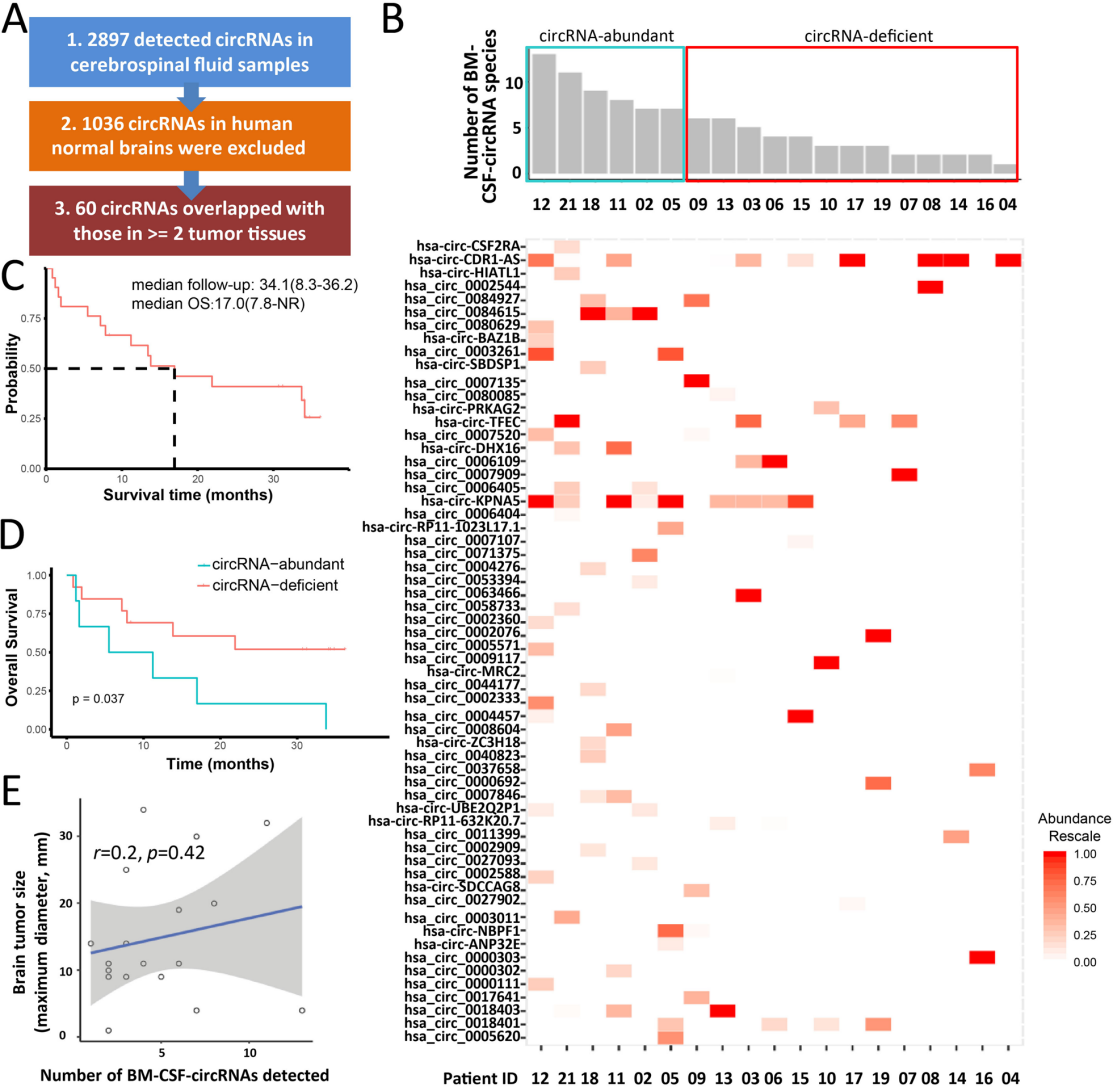
Characteristic of the sixty CSF-circRNAs and their association with the clinical outcome. **A** Flowchart for screening human lung cancer brain metastasis-related cerebrospinal fluid circRNAs. **B** The number (above) and expression abundance (below) of 60 identified cerebrospinal fluid circRNAs in each lung cancer brain metastasis patient. The intense of red color in the heatmap implicated the expression abundance of specific circRNA in the patient. **C** Overall survival of all patients enrolled. Survival curve was drawn by Kaplan-Meyer analysis to estimate median survival time. NR: not reached. **D** Kaplan–Meier overall survival curves of lung cancer brain metastasis patients according to 60 CSF circRNAs. **E** Correlation between the number of CSF-circRNAs and tumor size

A total of 2,897 circRNAs were identified in CSF from BM lung cancer patients. The percentage of circRNAs was the same as previously found in brain tissue [44] and ranged from 47 to 81% of the total CSF-circRNAs in each patient (Additional file 5: Table S6). Some criteria were applied to effectively screen for cancer-related CSF-circRNAs (Additional file 6: Figure S9). Initially, 1036 circRNAs previously reported in human normal brain tissue [44] were excluded. To exclude possible normal tissue circRNAs, only those circRNAs, which appeared in tumor tissues at least two patients, were kept for further analysis. Finally, sixty potentially cancer-related circRNAs were remained (Fig. 2A). The expression abundance of 60 selected circRNAs was identified in individual patient (Fig. 2B).

The median OS of total group was 17.0 months with a median 34.1 months follow-up (Fig. 2C). Patients were divided into CSF circRNA-abundant and CSF circRNA-deficient groups based on the top 25% (the cutoff is 7) of expressed circRNAs among 60 selected candidates. Patients in CSF circRNA-deficient group (≤ 7 of 60 selected circRNAs) had longer OS than CSF circRNA-abundant (> 7 of 60 selected circRNAs) (*p* = 0.037, in Fig. 2D). An insignificant positive correlation was observed between the number of detected candidates among 60 CSF-circRNAs and BM lesion size, which was the longest diameter of the largest lesion (Fig. 2E, Additional file 5: Table S5).

### Sixty CSF-circRNAs were associated with cell proliferation and differentiation pathways

**Fig. 3:**
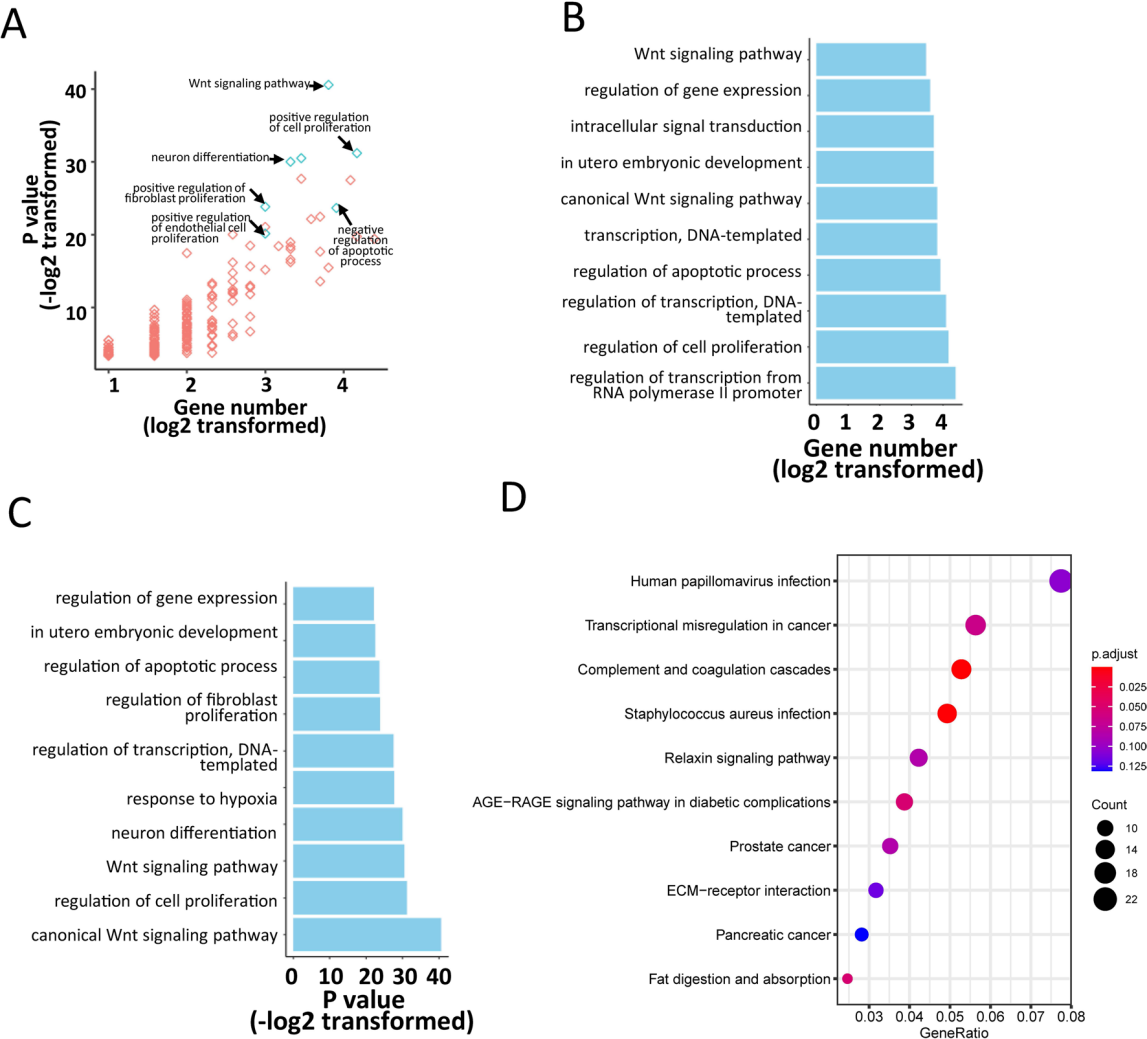
Function annotations and enrichment for the target genes of 60 CSF circRNAs. **A** KEGG pathway enrichment analyses of the target genes of co-expressed mRNA of 60 CSF circRNAs. **B** Cancer-associated pathway using DAVID function annotation for the 60 CSF-circRNAs targeted genes. The scatter plots demonstrated all features with p-value and gene counts. Positive regulation of cell proliferation and negative regulation of apoptosis were labelled. The detailed gene counts and p-value were shown in C, D. The vertical axis shows the annotated functions of the target genes. The horizontal axes show log2 transformed gene number and − log2 transformed p-value respectively. Only the most significantly enriched pathways were included

To study the biological functions of the sixty circRNAs, circRNA-miRNA-mRNA network (method) was constructed. Cancer-associated pathways enriched in target genes of the 60 CSF-circRNAs were analyzed by using DAVID (Fig. 3A). Majority of target genes were correlated with increased cell proliferation and differentiation and reduced apoptotic process. Both canonical [45] and non-canonical Wnt signaling [46] pathways, which have been known to support tumor metastasis [47, 48], had the most enrichment of target genes (Fig. 3B, C). KEGG pathway enrichment analyses were performed using target genes of mRNA co-expressed with the sixty CSF-circRNAs and the transcriptional misregulation in cancer was identified as one of the most enriched biological processes (Fig. 3D).

### Validation of selected WNT signalling pathway associated circRNAs

**Fig. 4:**
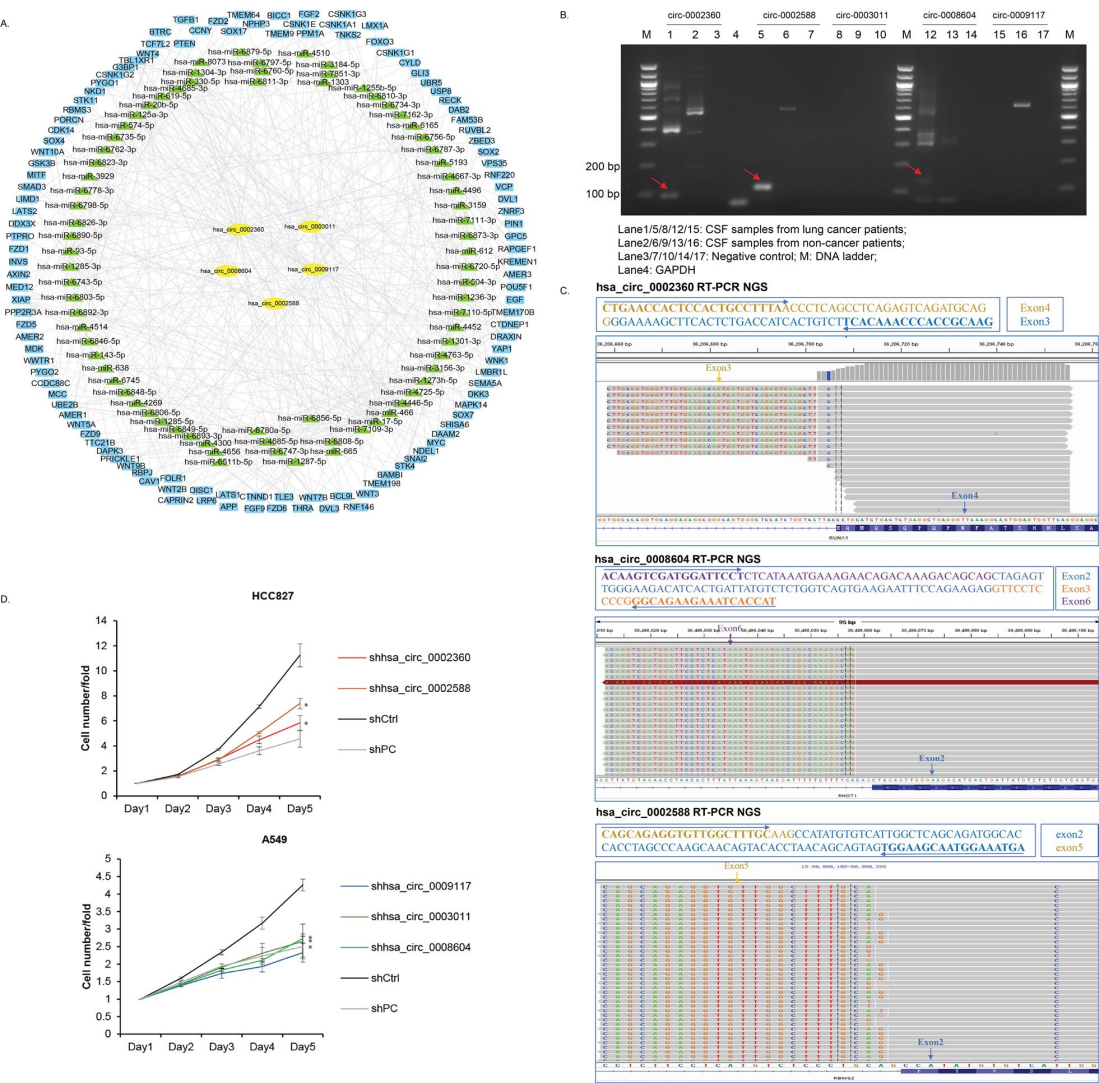
Validation of potential roles of selected circRNAs. **A** circRNA-miRNA-mRNA network of the five WNT-associated CSF-circRNAs. **B** Quantification of five circRNAs by RT-PCR and agarose gel electrophoresis. GAPDH was used as internal control. **C** NGS of circRNAs after RT-PCR. **D** Detection of cell growth by Celigo automated imaging and cell counting platform. Triple experiments were performed. Values were represented as mean ± SD. T-test was used for comparison. *: P < 0.05

Five of 60 candidates CSF-circRNAs, which were hsa_circ_0003011 (parental RFWD2), hsa_circ_0008604 (parental RHOT1), hsa_circ_0002360 (parental RUNX1), hsa_circ_0009117 (parental RERE), and hsa_circ_0002588 (parental RBMS2), were annotated with top-rank WNT signaling pathways in circRNA-miRNA-mRNA interacting network and were selected for further validation (Fig. 4A).

RT-PCR was performed to examine circRNA expression in CSF from another 10 BM lung adenocarcinoma patients and two non-cancer patients as normal control. It was showed that circ_0002360, circ_0002588 and circ_0008604 were only identified in CSF from lung cancer patients (Fig. 4B) and NGS confirmed their sequences. Circ_0003011 and circ_0009117 were not found in CSF from either cancer or non-cancer patients.

Lentiviral shRNA vectors were constructed targeting the five circRNAs (hsa_circ_0003011, hsa_circ_0008604, hsa_circ_0002360, hsa_circ_0009117, and hsa_circ_0002588) and A549 and HCC827 cell lines were infected. The levels of hsa_circ_0002360 and hsa_circ_0002588 significantly decreased with shRNA knock-down in HCC827 (Additional file 6: Figure S10A), and the proliferation of cells transfected with these shRNAs was greatly reduced (Fig. 4D). In A549 model, shRNAs significantly knocked down hsa_circ_0003011, hsa_circ_0008604 and hsa_circ_0009117 (Additional file 6: Figure S10A) while exhibited negative impact on cell proliferation (Fig. 4D, Additional file 6: Figure S10B, D) and invasion ability of A549 cells was also undermined (Additional file 6: Figure S10C).

## Discussion

Numerous reports have shown the potential roles of circRNAs in tumor metastasis [16, 18, 23, 24, 49, 50]. While most studies focused on tumor tissues, there are increasing interests in circRNAs analysis using liquid biopsies [50, 51]. Given the inaccessibility of brain tumors from metastatic lung cancer patients, the combination of circRNA and liquid biopsy would provide more information to the treatment management. The frequently renewed cerebrospinal fluid keeps intimate contact with brain malignancies [8, 9], may contain more tumor-specific molecules but fewer contaminations from blood cellular components than plasma [52], and thus may serve as a promising reservoir of BM-related circRNAs. However, little comparative analysis of circRNAs derive from tumor tissues, plasma and CSF has been reported in clinical settings. In this study, CSF-circRNAs gained more diversity compared to circRNAs from tumor and plasma across patients, indicating the inter-patient heterogeneity. A total of 60 potential prognosis-related CSF-circRNAs were identified and they are reasonably deemed to be BM-associated on the basis of fact that free circRNAs escaping from primary lung tumors to the brain metastases is strictly regulated by blood–brain barrier.

CircRNAs’ function is known to be more associated with their target genes rather than parental genes. Our results showed that the candidate target genes of the 60 circRNAs were significantly enriched in the Wnt signaling pathways, which were tightly associated with cancer development [47], maintenance of cancer stem cells [53], and metastasis [47]. The presence of *FZD2* and its ligand *WNT5A* (associated circRNAs: hsa_circ_0002360, hsa-circ-DHX16, hsa_circ_0002544, hsa_circ_0008604, hsa_circ_0009117) implied the enhanced epithelial to mesenchymal transition and cell migration [47]. In addition, *MYC* (associated circRNAs: hsa_circ_0002588, hsa_circ_0007909, hsa_circ_0008604), the well-known metastasis gene for lung cancer [54], was also identified. These data support the association between brain metastasis and the 60 circRNAs and guarantee future study on development and metastasis of lung cancer.

Five WNT-pathway related circRNAs including hsa_circ_0002360 (parental RUNX1), hsa_circ_0002588 (parental RBMS2), hsa_circ_0009117 (parental RERE), hsa_circ_0003011 (parental RFWD2), and hsa_circ_0008604 (parental RHOT1) were demonstrated to function in in vitro assays. As previously reported, hsa_circ_0002360 (parental RUNX1) overexpressed in lung adenocarcinoma and hsa_circ_000-2360/hsa-mir-3620-5p/PHF19 might interact in the progression of lung adenocarcinoma under cicrRNA-miRNA-mRNA networks [22]. Our results also showed that hsa_circ_0002360 (parental RUNX1) as one of 60 candidates circRNAs shared between CSF and tumor samples might function in the lung adenocarcinoma cell proliferation, migration and invasion.

Among the target genes of 5 validated circRNAs in Wnt pathway, many were identified to function in progression and metastasis of lung cancer. Wnt signaling is of great importance and it is common in lung cancer to have overexpression of Wnt-1/2/3/5a and other components (*e.g.* FZD, DVL, PORCN, TCF) [55, 56], which were predicted as the target genes for hsa_circ_0002360 (target genes: Wnt-2/3/5a/ FZD/DVL1/PORCN/TCF), hsa_circ_0003011 (target genes: FZD), hsa_circ_0008604 (target genes: Wnt-3/5a), hsa_circ_0009117 (target genes: Wnt-2/3/5a/FZD/DVL3/PORCN/TCF). In lung cancer, DVLs was reportedly to activate Wnt pathway [57] and brain metastasis had increased expression of DVL1 and DVL3 [58], which are potential targets of hsa_circ_0002360 and hsa_circ_0009117, respectively.

There were some limitations in this study. The validation experiments were preliminary and the function of these circRNAs were not comprehensively demonstrated. Due to the small size of group of patients, the survival analysis results of circRNAs are not convincing enough. However, further studies are guaranteed on the basis of our study.

Collectively, this study provided a leading and fundamental circRNAs profiles of lung cancer brain metastasis by using CSF.

## Conclusions

Our data showed that the 60 circRNAs might play important roles in regulating biological functions and cancer-related signaling pathways and might be associated with cell proliferation and invasion process. CSF-circRNAs might be promising biomarkers in the lung cancer patients with brain metastases.

## Supplementary Information

**Additional file 1: Table S1.** QC information.

**Additional file 2: Table S2.** FPKM of all samples.

**Additional file 3: Table S3. **circRNA-miRNA-mRNA.

**Additional file 4: Table S4.** circRNA-coexpressed mRNA symbol.

**Additional file 5: Table S5.** Clinical data pertaining to brain/leptomeningeal metastasis. **Table S6. **CSF circRNAs in this study and normal brain circRNAs previously reported.

**Additional file 6. **Additional figures S1 to S10.

## Data Availability

The data used and/or analyzed during the current study are available from the corresponding author on reasonable request.

## Abbreviations

circRNAs: Circular RNAs
ncRNAs: Noncoding RNAs
BM: Brain metastasis
CSF: Cerebrospinal fluid
ADC: Adenocarcinoma
TMM: Trimmed means of M-values
DAVID: Database for annotation, visualization and integrated discovery
PC: Positive control
PBS: Phosphate-buffered saline
ctDNA: Circulating tumor DNA
OS: Overall survival
HR: Hazard ratio
PFS: Progression-free survival
