# Supplementary material for: Clinical utility of cerebrospinal fluid-derived circular RNAs in lung adenocarcinoma patients with brain metastases
Source: J Transl Med. 2022 Feb 5;20:74. doi: 10.1186/s12967-022-03274-1 (PMC8818222; doi:10.1186/s12967-022-03274-1)
Supplement: Supplementary file 5 — Additional file 5: Table S5. Clinical data pertaining to brain/leptomeningeal metastasis. Table S6. CSF circRNAs in this study and normal brain circRNAs previously reported. [file 12967_2022_3274_MOESM5_ESM.docx]

**Table S5. Clinical data pertaining to brain/leptomeningeal metastasis**

|  | | | | | | | | | | | | |  | |
| --- | --- | --- | --- | --- | --- | --- | --- | --- | --- | --- | --- | --- | --- | --- |
| Patient | BM lesion size* (mm) | BM lesion number | CSF cytology | Genomic alterations in tumor tissue** | LMC/BM manifestation  *** | LMC diagnosed by MRI | Subsequent Treatment  **** | Subsequent treatment line setting | | Non-cranial metastatic organs | | OS after CSF sampling (months) | | |
| 1 | NA | NA | NA | EGFR L858R+T790M, TP53mut | - | - | Osimertinib | 2 | | bone, lung | | NA | | |
| 2 | 30 | 4 | + | EGFR exon 19del+T790M, TP53mut | + | - | WBI, BSC | 3 | | pericardium, bone, cervical lymph nodes | | 5.5 | | |
| 3 | 9 | 3 | - | EML4-ALK rearrangement | + | - | WBI, BSC | 3 | | adrenal gland, peritoneal lymph nodes | | 0.8 | | |
| 4 | 14 | 4 | - | EGFR L858R+T790M | - | - | SRS of BM, osimertinib | 2 | | lung, pleura, bone | | 36.2 | | |
| 5 | 4 | 4 | - | EGFR  exon 19 del | - | - | Paclitaxel +CBP+Bev | 4 | | Bone, pleura, liver | | 33.8 | | |
| 6 | 11 | 3 | - | EML4-ALK rearrangement | - | - | Crizotinib | 1 | | lung, bone | | 30.7 | | |
| 7 | 11 | 4 | - | EGFR L858R, ERBB2 amplification, TP53mut | - | - | Afatinib+Bev | 2 | | bone, lung, adrenal gland | | 13.8 | | |
| 8 | 9 | 3 | - | TSN-ALK rearrangement, TP53mut | - | - | Foritinib  (SAF-189s) | 3 | | lung, liver | | 34.1 | | |
| 9 | 19 | 1 | - | EGFR L858R+T790M | - | + | Osimertinib | 2 | | No | | 34.3 | | |
| 10 | 9 | 2 | - | EGFR exon 19del+T790M | - | - | BSC | 2 | | Lung | | 1.9 | | |
| 11 | 20 | 1 | - | EGFR exon 19del+T790M | + | - | SRS of BM, osimertinib | 2 | | No | | 16.9 | | |
| 12 | 4 | 4 | + | EGFR exon19del + (T790M + C797S in cis) and (T790M + C797S in trans) | + | - | Erlotinib+ osimertinib | 6 | | pleura, peritoneal lymph nodes, adrenal gland | | 1.6 | | |
| 13 | 11 | 2 | - | EGFR L858R+T790M, TP53mut | - | + | Osimertinib | 2 | | lung | | 21.9 | | |
| 14 | 10 | 3 | - | EGFR exon19 del, TP53mut | - | - | SRS of BM, gumetinib (SCC244) | 3 | | | pleura, axillary lymph nodes | 34.8 | | |
| 15 | 34 | 2 | - | EGFR exon 19del+T790M, TP53mut | + | - | Resection of BM, Osimertinib | | 2 | | bone | 31.2 | | |
| 16 | 1 | 3 | - | EGFR L858R+T790M, TP53mut | - | - | Osimertinib | | 4 | | pleura, bone | 7.8 | | |
| 17 | 14 | 3 | - | KRAS G12C, TP53mut | - | - | Pem+Bev | | 2 | | liver, bone | 7.2 | | |
| 18 | NA | 3 | + | EGFR exon 20ins (M766delinsMASV), TP53mut | + | + | BSC | | 2 | | pleura, pericardium | 1.2 | | |
| 19 | 25 | 1 | - | EGFR L858R, TP53mut | + | - | Erlotinib | | 1 | | No | 8.4 | | |
| 20 | NA | 5 | NA | EGFR L858R+T790M, TP53mut | + | - | Osimertinib | | 2 | | lung, bone, peritoneum | NA | | |
| 21 | 32 | 1 | - | EGFR exon 19del, TP53mut | - | - | Pem+CBP+Bev | | 3 | | pleura | 11.2 | | |
| BM, brain metastasis; CSF, cerebrospinal fluid; LMC, leptomeningeal carcinomatosis; MRI, magnetic resonance imaging; NA, not available; EGFR, epidermal growth factor receptor; WBI, whole brain irradiation; BSC, best support care; ALK, anaplastic lymphoma kinase; KRAS, Kirsten rat sarcoma viral oncogene homologue; SRS, stereotactic radiosurgery; Pem, pemetrexed; CBP, carboplatin; Bev, bevacizumab; +: positive; -: negative; OS: overall survival.  *BM lesion size is the longest diameter of the largest lesion. Patient 18 had appearance of LMC in brain MRI with three small plaque-like lesions without definite border in left occipital lobe and cerebellum and tumor size was therefore not listed. Patient 20 had a brain MRI examination in another hospital and the report showed five BM lesions without the description of tumor size. Patient 1 had no BM lesion by MRI or neurological symptoms at the time of tissue and blood collection, however, a month later BM appeared in MRI accompanied with headache. Patient1 and patient 20 didn’t undergo CSF sampling.  ** Genomic alterations refer to next-generation sequencing (NGS) of genomic DNA in tumor tissue. NGS of cell-free DNA in blood and CSF is not shown.  *** LMC and BM to some extent share similar symptoms, which included headache, confusion, cognitive impairment, visual changes, speech problems, paralysis, dysequilibrium, psychiatric disorders, seizures, etc. Five patients (P02, P09, P12, P13, P18) were considered to have leptomeningeal carcinomatosis (LMC). P12 and P18 displayed positive CSF cytology accompanied by neurological symptoms, which included headache, confusion, cognitive impairment and psychiatric disorders. Patient 18 also showed positive in brain MRI. P09 and P13 were diagnosed with LMC by brain MRI. Both patients showed no LMC manifestation and negative CSF cytology. Patient 9 had LMC diagnosis by brain MRI, which showed local meningeal thickening in parietal lobe with obvious enhancement. Patient 13 had brain MRI revealing nodular lesion in left postcentral gyrus with central spotty, patchy necrosis, annular enhancement, and surrounded by edema signal. Patient 3, 11, 12, 15, 19, 20 had only neurological symptoms.  ****Refers to treatment regimen patient received after sampling | | | | | | | | | | | | | |  |

| **Table S6. CSF circRNAs in this study and normal brain circRNAs previously reported.** | | | |
| --- | --- | --- | --- |
| Patient | Number of normal CSF circRNAs | Number of total CSF circRNAs | Percentage  (normal /total CSF circRNAs) |
| 02 | 252 | 327 | 0.770642 |
| 03 | 117 | 202 | 0.579208 |
| 04 | 69 | 85 | 0.811765 |
| 05 | 118 | 155 | 0.76129 |
| 06 | 81 | 113 | 0.716814 |
| 07 | 63 | 85 | 0.741176 |
| 08 | 55 | 72 | 0.763889 |
| 09 | 132 | 193 | 0.683938 |
| 10 | 46 | 92 | 0.500000 |
| 11 | 113 | 163 | 0.693252 |
| 12 | 201 | 306 | 0.656863 |
| 13 | 151 | 197 | 0.766497 |
| 14 | 65 | 86 | 0.755814 |
| 15 | 119 | 152 | 0.782895 |
| 16 | 161 | 341 | 0.472141 |
| 17 | 102 | 133 | 0.766917 |
| 18 | 455 | 650 | 0.700000 |
| 19 | 68 | 82 | 0.829268 |
| 21 | 207 | 259 | 0.799228 |
