# Supplementary material for: Clinical utility of cerebrospinal fluid-derived circular RNAs in lung adenocarcinoma patients with brain metastases
Source: J Transl Med. 2022 Feb 5;20:74. doi: 10.1186/s12967-022-03274-1 (PMC8818222; doi:10.1186/s12967-022-03274-1)
Supplement: Supplementary file 6 — Additional file 6. Additional figures S1 to S10. [file 12967_2022_3274_MOESM6_ESM.docx]

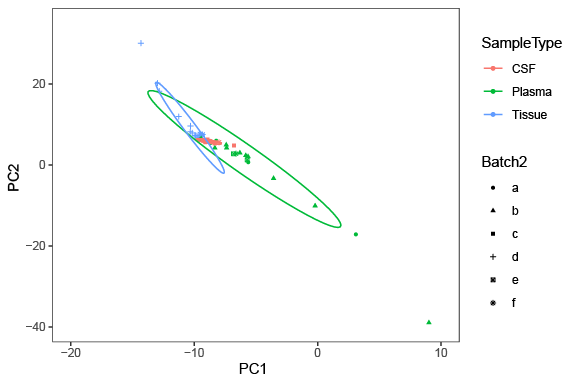


Figure S1. Principal component analysis(PCA) of circRNAs(CSF, plasma, tissue) from different batches(a,b,c,d,e,f). The FPKMs of the circRNAs were used for PCA.

Figure S2. Distribution of the expression abundance of circRNAs in 3 different types of samples (*e.g.* cerebrospinal fluid, plasma, tumor tissues), divided by patients.

Figure S3. Distribution of the ratio of expression abundance of circRNAs/parental mRNAs in 3 different types of samples (*e.g.* cerebrospinal fluid, plasma, tumor tissues), divided by patients.

Figure S4A. Correlation of the expression abundance between circRNAs and parental mRNAs in cerebrospinal fluid, divided by patients.

Figure S4B. Correlation of the expression abundance between circRNAs and parental mRNAs in plasma, divided by patients.

Figure S4C. Correlation of the expression abundance between circRNAs and parental mRNAs in tumor tissues, divided by patients.

Figure S5. Overlapping of the number of detected circRNAs in 3 different types of samples (*e.g.* cerebrospinal fluid, plasma, tumor tissues), divided by patients.


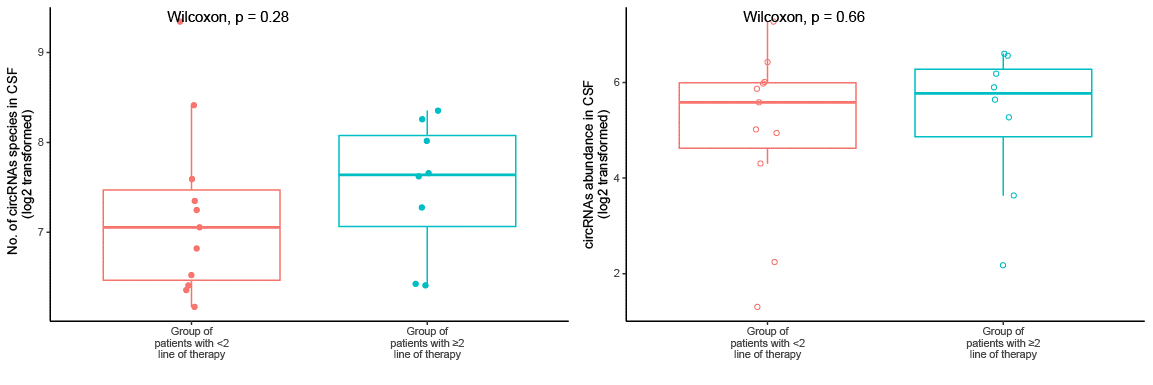


Figure S6. Comparison of circRNA species (A) and expression abundance (B) in CSF between groups receiving different number of prior therapies. No.：number.

Figure S7. Correlation between the number (left)/abundance (right) of total circRNAs and variant allele fraction (VAF) values in different samples (from top to bottom: cerebrospinal fluid, plasma, tumor tissues). VAF was used to indicate the ctDNA concentration in each sample. The horizontal axes showed log2 transformed circRNAs number and expression respectively. rpkm: per Million mapped reads, was a normalized unit of transcript expression.


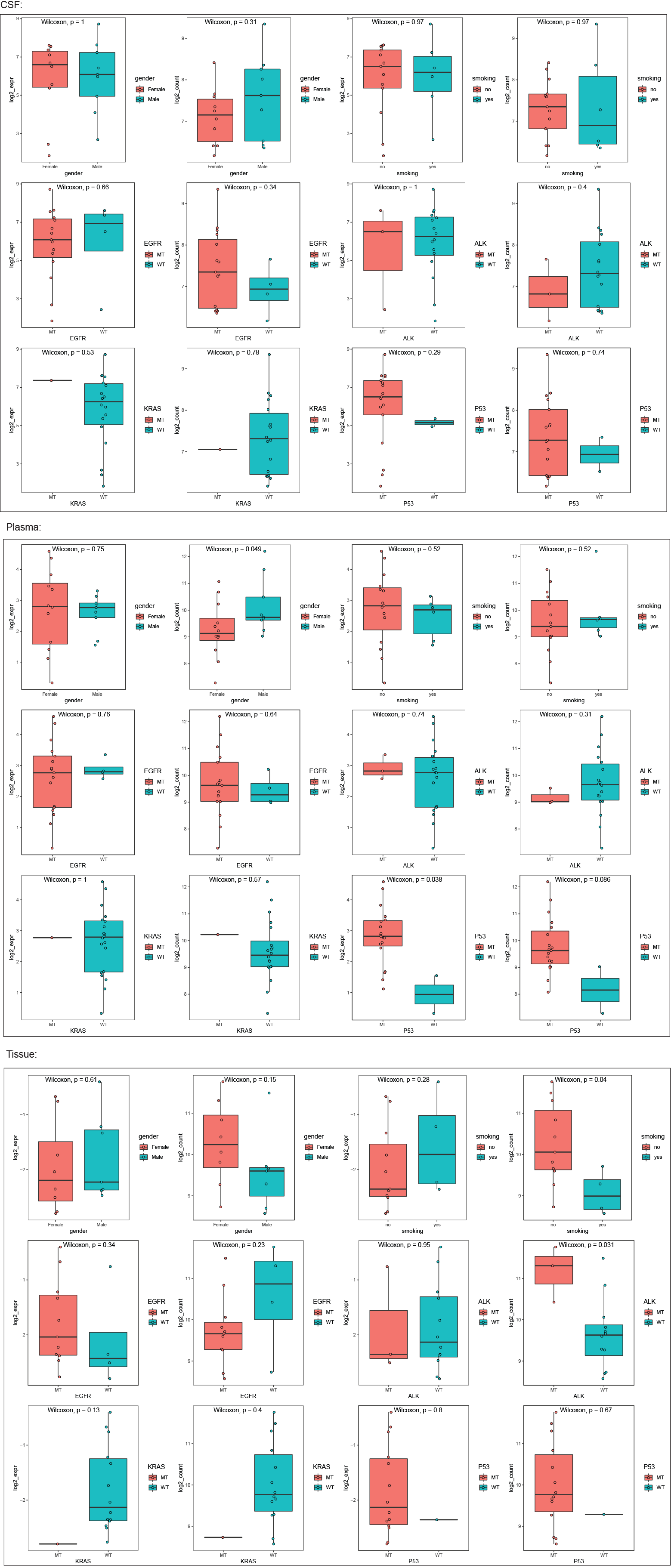


Figure S8. The number of circRNA species or circRNA abundance in different samples from lung cancer BM+ patients with distinct molecular or clinical features. Molecular features analyzed included *EGFR*, *ALK, KRAS, TP53* alterations. Clinical features included smoking, gender.


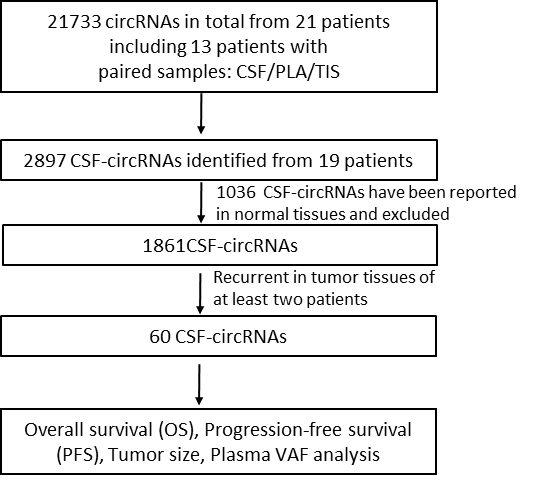


Figure S9. The flowchart of circRNAs filtering in the present study. A total of 21733 circRNAs were detected from 3 different types of samples (2897 in cerebrospinal fluid, 13566 in plasma, 10225 in tumor tissues) and subjected to downstream filtering. PLA: plasma; TIS: tumor tissue.

**
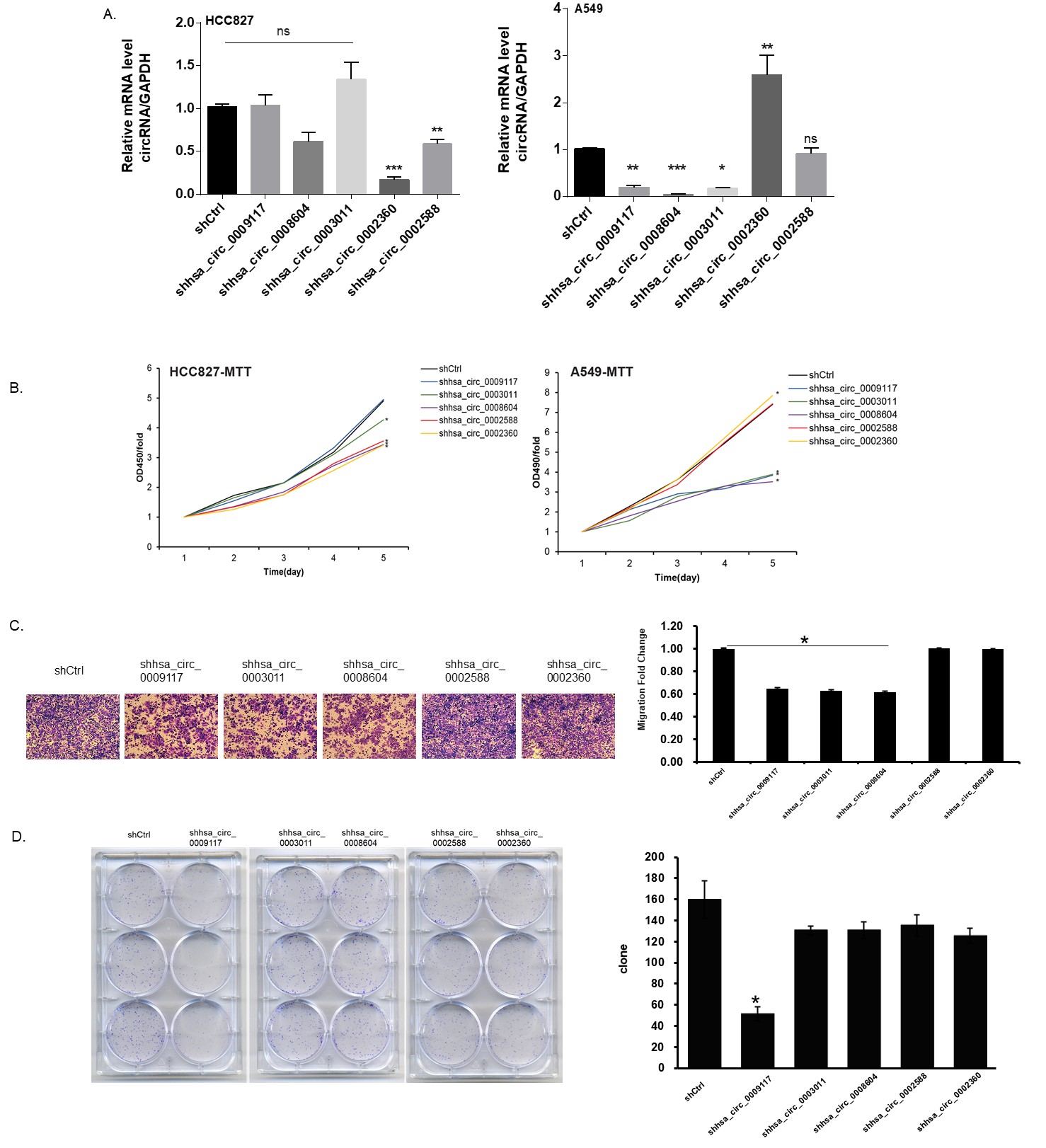
**

Figure S10. Experimental validation of potential roles of selected circRNAs using HCC827 and A549 cell line. (A)Knock down efficiency of shRNAs targeting different circRNAs were performed in HCC827 and A549 cell line using real-time PCR. (B) cell proliferation assay in HCC827 and A549. Proliferation of cells with different circRNAs knockdown was measured using MTT assay. All experiments were performed in duplicates and repeated at least three times independently. (C) Transwell assay in A549 cell (magnification: x100). (D) Colony formation assay in A549 cell. Triple experiments were performed. Values were represented as mean ± SD. T-test was used for comparison. *: P<0.05; **: P<0.01; ***: P<0.001.
